# Fgf3 and Fgf10a Work in Concert to Promote Maturation of the Epibranchial Placodes in Zebrafish

**DOI:** 10.1371/journal.pone.0085087

**Published:** 2013-12-17

**Authors:** Matthew N. McCarroll, Alex V. Nechiporuk

**Affiliations:** Department of Cell and Developmental Biology, Oregon Health and Science University, Portland, Oregon, United States of America; Texas A&M University, United States of America

## Abstract

Essential cellular components of the paired sensory organs of the vertebrate head are derived from transient thickenings of embryonic ectoderm known as cranial placodes. The epibranchial (EB) placodes give rise to sensory neurons of the EB ganglia that are responsible for relaying visceral sensations form the periphery to the central nervous system. Development of EB placodes and subsequent formation of EB ganglia is a multistep process regulated by various extrinsic factors, including fibroblast growth factors (Fgfs). We discovered that two Fgf ligands, Fgf3 and Fgf10a, cooperate to promote EB placode development. Whereas EB placodes are induced in the absence of Fgf3 and Fgf10a, they fail to express placode specific markers Pax2a and Sox3. Expression analysis and mosaic rescue experiments demonstrate that Fgf3 signal is derived from the endoderm, whereas Fgf10a is emitted from the lateral line system and the otic placode. Further analyses revealed that Fgf3 and Fgf10a activities are not required for cell proliferation or survival, but are required for placodal cells to undergo neurogenesis. Based on these data, we conclude that a combined loss of these Fgf factors results in a failure of the EB placode precursors to initiate a transcriptional program needed for maturation and subsequent neurogenesis. These findings highlight the importance and complexity of reiterated Fgf signaling during cranial placode formation and subsequent sensory organ development.

## Introduction

In the developing vertebrate head, essential cellular components of the paired sensory organs originate from anatomically distinct structures consisting of neurogenic epithelium called cranial placodes. Cranial placodes are morphologically defined as transient ectodermal thickenings, with columnar or pseudostratified epithelial cell morphology. Through physical interactions with neighboring tissues (i.e. neural crest, mesoderm and endoderm) and in response to extrinsic signals, cells of the cranial placodes delaminate and/or invaginate to form structures as diverse as the optic lens, the otic vesicle, and neurons of the cranial ganglia. Epibranchial (EB) placodes (comprised of the facial, glossopharyngeal, and vagal) give rise to sensory neurons of the EB ganglia. EB neurons act as a relay for information from the sensory organs (e.g. taste buds of the gustatory system, baroreceptors of the heart, and sensory enteric nerves of the gut) to the CNS [1,2].

Several lines of evidence from multiple vertebrate species indicate that placode development is a multi-phase process [3]. At the end of gastrulation, all cranial placode precursors reside in a horseshoe shaped domain known as the pan-placodal ectoderm [4–7]. Shortly thereafter, regional cell fate specification begins. The most posterior portion of the pan-placodal ectoderm, the posterior placodal area (PPA), will undergo different stages of specification, induction and morphological changes to ultimately give rise to the otic and EB placodes [8–10]. In aquatic vertebrates the PPA will also give rise to the lateral line system [11,12]. 

Recent studies have illustrated an important interaction between the developing cranial placodes and the cranial neural crest at multiple stages of EB placode development. These two tissues interact to orchestrate cellular movements by providing both guidance cues and contact inhibition of locomotion, recently described as a chase-and-run behavior where the NC will chase early placode precursor cells which in turn run from the NC, resulting in proper segregation of the cranial placodes [13]. Once EB placodes form, the cranial neural crest is necessary for appropriate condensation of the cranial ganglia. In zebrafish, genetic ablation of the neural crest results in disorganization and reduction of the placode-derived cranial ganglia [14]. A recent study in both chick and mouse has identified a neural crest derived structural corridor that is necessary for the migrating placodal neuroblasts to reach their final destination and form properly positioned ganglia [15]. Altogether, these studies provide strong evidence for the importance of interactions between placode and neural crest populations to achieve correct migration and patterning of the cranial placodes and subsequent ganglia.

Multiple transcription factors expressed during various stages play distinct roles in cranial placode development. Foxi1 is a winged helix transcription factor that is important for development of the otic and EB placodes; this factor is thought to impart placodal competence to the ectoderm [16–19]. While Foxi1 is a broadly expressed competence factor, another PPA factor, Sox3 is more specifically expressed in the otic anlage and is among the first known factor to be detected in the EB placode precursors. Sox3 expression is also maintained in the mature EB placodes [20,21]. Pax2a is similarly expressed in the otic anlage, and later in maturing EB placodes [12]. Finally cells of the fully mature EB placodal ectoderm express basic helix-loop-helix factor Neurogenin1 (Neurog1), which is required for neurogenesis [17,22]. Neurog1 is transiently upregulated in placodal cells as well as delaminating neuroblasts. Once migrating neuroblasts condense into EB ganglia, they begin to express another neurogenic factor, paired-like homeobox 2b (Phox2b) that marks differentiated EB neurons [23,24]. 

Signaling through fibroblast growth factors (Fgfs) are essential during multiple stages of placode progression from the early homogeneous precursor stage to formation of discrete placodes and subsequent neurogenesis [10,24]. In zebrafish, mesoderm-derived expression of Fgf3 and Fgf8 are required for the specification of the early PPA, as a combined loss of these factors results in an abnormal distribution of *foxi1* and absence of the otic and EB placodes [17,25–25–27]. At later stages, endoderm-derived Fgf3 is required again for neurogenesis of the glossopharyngeal and three small vagal ganglia, but the facial and large vagal ganglia appear unaffected in *fgf3* deficient embryos [24]. Interestingly the EB ganglia develop at the dorsal aspect of the branchial arches, and Fgf3 is also important for the proper development of the endodermal pouches [24,28]. These pouches are populated with chondrogenic NC cells that give rise to mature branchial arches. In *fgf3* mutants, NC derived chondrogenic precursors migrate to their destination, however they are not properly maintained, do not undergo a chondrogenic program and will eventually undergo cell death [28]. These studies illustrate the complexity of tissue interactions and convergent signaling pathways involved during placode development and subsequent neurogenesis. However, after the initial specification of the PPA precursors, it is currently unknown what specific signals are needed for the proper development of the EB placodes.

Our data involving either disruption or over-activation of Fgf signaling provide strong evidence that an Fgf is required for EB placode maturation [12,17]. When Fgf receptor signaling is globally inactivated between 12 and 22 hpf EB placode markers are lost or severely disrupted, while other placode derived structures (like the otic vesicle) remain relatively intact. Close analysis of Fgf ligand expression revealed that two candidates, Fgf3 and Fgf10a, were temporally and spatially positioned for proper maturation and subsequent neurogenesis of the EB placodes. We find that injection of *fgf10a*-MO (morpholino) into fgf3^-/-^ mutant embryos resulted in loss of the facial placode, a nearly complete loss of the glossopharyngeal and vagal placodes at 24 hpf, and an absence of the respective ganglia at 72 hpf. We also find that Fgf3/10a deficient embryos exhibit a loss of anterior otic identity and a stalling of the anterior lateral line. Furthermore we have identified the endoderm and the lateral line systems as the tissue sources of Fgf3 and Fgf10a, respectively, during this critical period. We determine that these factors do not control placode cell morphology, and only partially disrupt the placode NC interaction, but are required for EB placode precursors to express Pax2a and Sox3 necessary for cellular entry into a neurogenic program.

## Materials and Methods

### Fish strains, maintenance, and Transgenesis

Breeding and maintenance of zebrafish were performed as described [29] and staged in hours post fertilization (hpf) [30]. The following transgenic and mutant lines were used for this study: *AB, Tg(*pax2a:GFP*)^*e1*^ [31], TgBAC(*phox2b:EGFP*)^37^ [17], *lia* ^t26121^ [32] TgBAC(*foxi1:d2EGFP*)^*nl11*^, Tg(*pax2a:Kaede*)^*nl12*^, TgBAC(*neurog1:DSRed*)^*nl6*^ [33], *Tg*(*sox10*(*7.2*)*:mrfp*)^*vu234*^ [34]. Heterozygous *lia* ^t26121^ were used to generate homozygous mutant offsprings that were identified by genotyping with the following primers: Fgf3 F: 5’-CCCATGAACTCATCTCGTACC, Fgf3 R: 5’-GCTTCTTGGATCCGAGTTTG. 

### Whole-mount in-situ hybridization and immunostaining

Whole-mount immunostaining and in-situ hybridization were performed as described [22]. The following antibodies and riboprobes were used: α-Pax2a (1:100, Covance), anti beta-Catenin (1:100, The Developmental Studies Hybridoma Bank, Iowa), *fgf3* [35], *fgf10* [36], *pax5 [37], sox3* [38], *eya1* [39], *foxi1* [16]. Whole-mount fluorescent images were obtained using an Olympus FV1000 confocal microscope. Brightfield images were acquired with an AxioImager Z1 compound microscope and HRc digital camera (Zeiss). Assembly of Z-stack images was performed using ImageJ [40]. Brightness and contrast were adjusted using Photoshop (Adobe).

### Morpholino Microinjections

Antisense morpholino oligonucleotides (MO) were obtained from GeneTools (Corvalis, OR), diluted to working concentrations in H_2_O and injected into TgBAC(*phox2b:EGFP*)^37^, TgBAC(*foxi1:d2EGFP*)^*nl11*^, TgBAC(*neurog:dsRED*)^*nl6*^ *, Tg*(*sox10*(*7.2*)*:mrfp*), and *lia* ^t26121^ embryos: *fgf3*-MO (5'-CATTGTGGCATGGCGGGATGTCGGC [25]; *fgf10-*MO (5’-GCTTTACTCACTGTACGGATCGTCC [41]; *cas-*MO (5’-GCATCCGGTCGAGATACATGCTGTT [42]. Efficacy of *fgf3* and *fgf10-*MO was assessed by fusion of the otoliths at 2 dpf [32] and loss of pectoral fins at 4 dpf [43] respectively.

### Tissue sections

TgBAC(*foxi1:d2EGFP*)^*nl11*^ embryos were injected with *fgf3* and *fgf10a*-MO were allowed to develop to 24 hpf and then fixed and probed with anti beta-Catenin. Embryos were then processed and cryosectioned as previously described [14]. Sections were counterstained with DAPI and fluorescent images were obtained using an Olympus FV1000 confocal microscope.

### Transplantation and bead experiments

Donor *AB embryos were microinjected at the one-cell stage with 10 kD fluorescein (Invitrogen) in 0.2M KCl. Embryos were dechorinated and allowed to develop to sphere and shield stage for donors and wild-type hosts, respectively. Twenty to 30 donor cells were transplanted into the presumptive placodal domain of *fgf3/10*-MO injected host embryos [44]. Mosaic embryos were fixed at 24 hpf and immunostained for Pax2a. Heparin coated (5 mg/ml), polystyrene beads (Polysciences, Philadelphia) were incubated with either 250 µg/ml of recombinant mouse Fgf8 protein (R&D Systems, Minnesota) or 0.5% BSA (Fisher Scientific) in PBS. Following incubation, beads were rinsed in PBS and implanted into 12-14 hpf wild-type embryos near the site of presumptive facial placodal precursors. Embryos were fixed at 24 hpf and immunostained for Pax2a.

## Results and Discussion

### Local Fgf activity is sufficient to expand the facial placode

We have previously demonstrated that Fgf activity is required for EB placode development during the second half of segmentation [12,17]. Fgf target genes, *pea3* and *erm*, are expressed in the placodal ectoderm at 13 hpf [24] and *pea3* has also been detected at 24 hpf [24]. Loss of Fgf signaling between 12 and 22 hpf largely disrupts EB placode development, without affecting gross morphology of the otic vesicle [17]. In addition, global activation of Fgf signaling after 12 hpf can expand the EB placode domain [12]. In this study, we asked whether local activation of Fgf signaling is sufficient to enlarge the EB placodes. To accomplish this we unilaterally inserted recombinant Fgf8- or BSA- (control) soaked heparin beads into 12 hpf embryos near the site of early EB precursors. Embryos were assayed for placodes at 24 hpf using the anti-Pax2a antibody. Instances with Fgf8 beads near the site of the facial placode showed a significant increase in the number of Pax2a+ cells (Figure S1A,B,F) compared to contralateral control side (Figure S1C,F), whereas BSA beads showed no change in placodal cell numbers (Figure S1D-F). These data indicate that the EB placodes display active Fgf signaling and that local activation of Fgf signaling after 12 hpf is sufficient to significantly expand the EB placodes. 

### Fgf3 and Fgf10a are expressed during epibranchial placode formation

**Figure 1:**
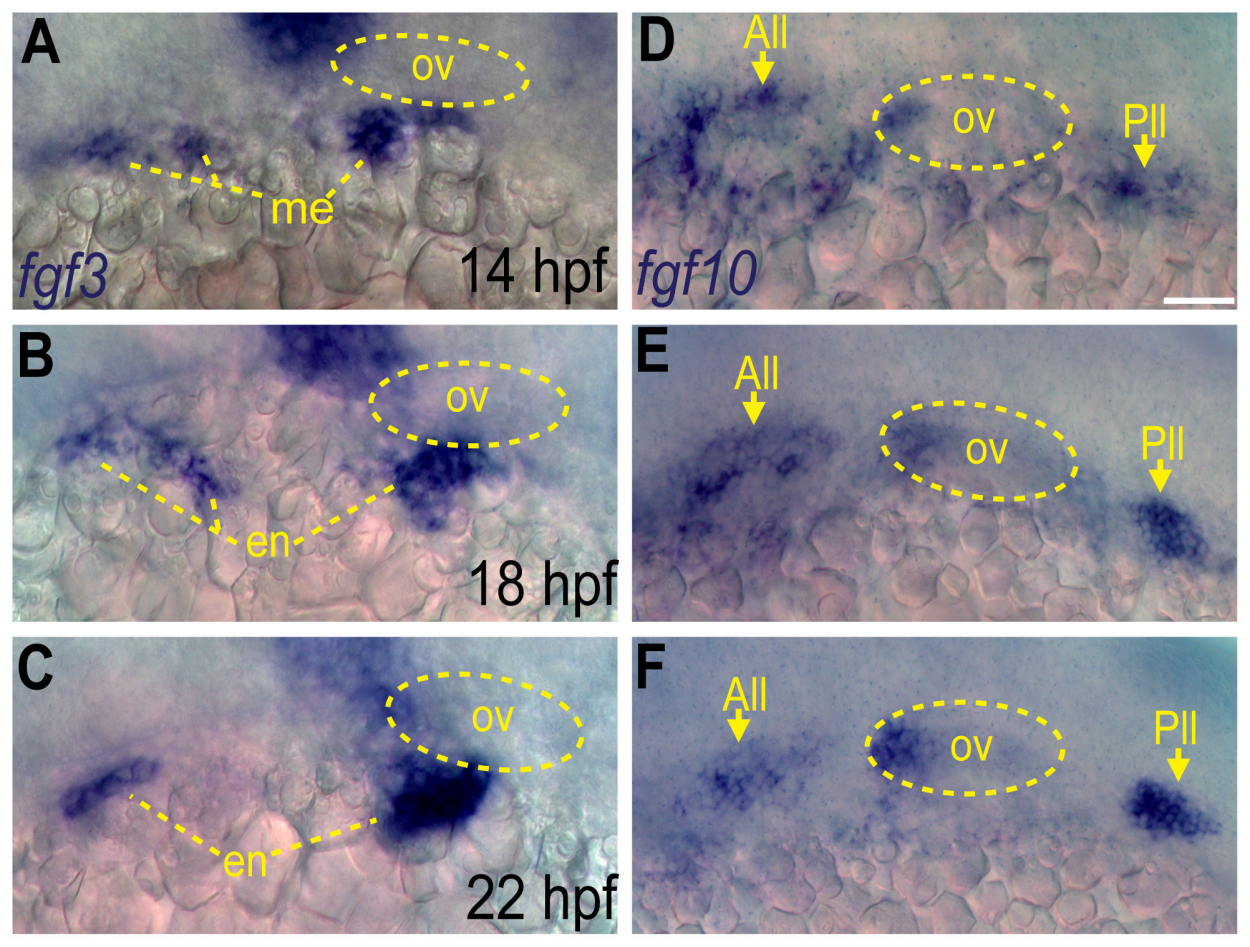
Fgf3 and Fgf10a are expressed during epibranchial placode formation. (**A**-**C**) In situ hybridization reveals presence of fgf3 transcript in the mesoderm at 14 hpf (A), and then in the endoderm at 18 (B) and 22 hpf (C). (**D**-**F**) In situ hybridization reveals presence of *fgf10a* transcript in the anterior and posterior lateral line (arrows) and the anterior portion of the otic vesicle at 14 (D),18 (E) and 22 hpf (F). Otic vesicle is outlined by a dotted line in (A-F). Abbreviations: ov, otic vesicle; me, mesoderm; en, endoderm; All, anterior lateral line; Pll, posterior lateral line. Scale bar: 50 µm.

Our data indicate that Fgf signaling between 12 and 22 hpf is necessary and sufficient for EB placode development [12,17], however the actual Fgf ligand(s) responsible for this activity are unknown. To determine the specific Fgfs involved in EB placode development, we searched for Fgf ligands expressed in the proximity of developing EB placodes between 12 and 22 hpf. *fgf3* is expressed in the mesoderm at 14 hpf, and then in the endoderm at later times (Figure 1A-C and [24,28]). Another Fgf ligand, *fgf10a*, was expressed in the anterior portion of the developing otic vesicle, anterior lateral line and posterior lateral line during these time-periods (Figure 1D-F). Thus, both Fgf3 and Fgf10a are expressed in the proximity of developing EB placodes during the critical time window, suggesting their requirement for the development of the EB placodes [12,17]. 

### Fgf3 and Fgf10a are required for maturation of epibranchial placodes and development of the epibranchial ganglia

**Figure 2:**
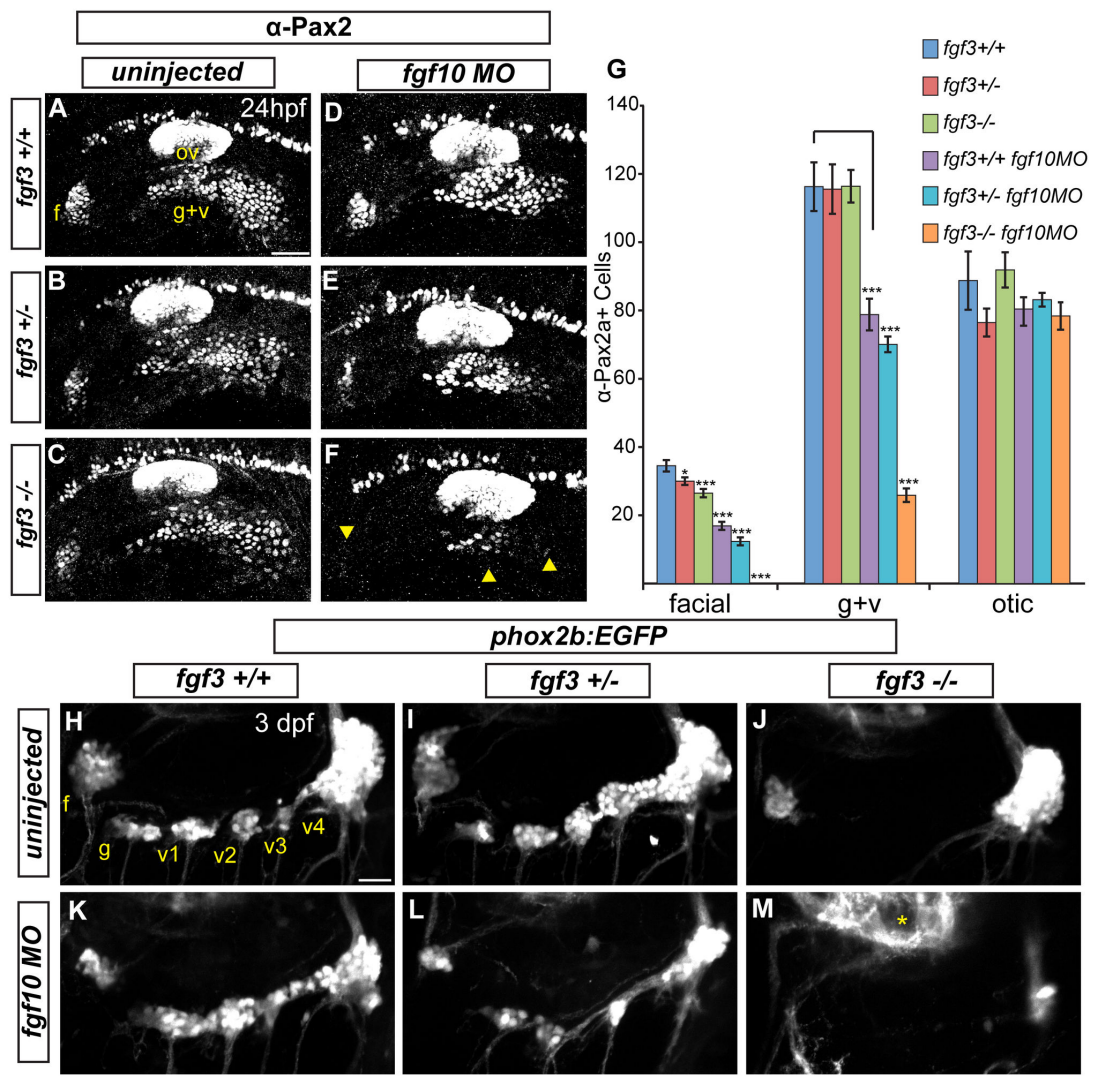
Fgf3 and Fgf10a are required for maturation of epibranchial placodes and development of the epibranchial ganglia. (**A**-**C**) Confocal projections showing Pax2a expression in wild-type (A) fgf3+/- (B) and fgf3-/- (C) embryos at 24 hpf. (**D**-**F**) Confocal projections showing Pax2a expression in 24-hour old wild-type (D) fgf3+/- (E) and fgf3-/- (F) embryos injected with *fgf10a*-MO. Note the significant loss of Pax2a expression in the EB placodes (F; arrowheads). (**G**) Pax2a+ cell number in the facial, glossopharyngeal/vagal, and otic placodes for conditions in (A-F). Note the complete loss of Pax2a+ cells in the facial placode and a 4.5 fold reduction in the glossopharyngeal/vagal placode in *fgf3-/-*;*fgf10a*-MO embryos (ANOVA multiple comparison with Bonferroni’s correction; *P<0.05; ***P<<0.001; Error bars: standard error of mean; n=11 embryos per condition). (**H**-**M**) Confocal projections of 3 dpf wild-type (H), fgf3+/- (I) and fgf3-/- (J) embryos and wild-type (K), fgf3+/- (L) and fgf3-/- (M) embryos injected with *fgf10a*-MO. All embryos contain TgBAC(phox2b:EGFP) which marks EB ganglia. Note the loss of EGFP expression in the glossopharyngeal and three small vagal ganglia in *fgf3-/-* embryos (J) and the complete loss of EGFP expression in all EB ganglia in fgf3-/-;*fgf10a*-MO with the exception of a few EGFP+ cells in the region of the large vagal ganglion (M). Asterisk marks hindbrain neurons also expressing TgBAC(phox2b:EGFP). Abbreviations: f, facial placode (A) or facial ganglia (H); ov, otic vesicle; g+v, glossopharyngeal/vagal placode; g, glossopharyngeal ganglia; v1-v3, small vagal ganglia 1-3; v4, large vagal ganglion. Scale bars: 50 µm (A); 25 µm (H).

Expression profiles of Fgf3 and Fgf10a indicated that these ligands could be involved in EB placode development. To test this, we injected the previously characterized *fgf10a-*MO, into embryos derived from heterozygous *fgf3+/-* crosses. At 24 hpf, we observed a slight reduction of Pax2a expression in the EB placodes of *fgf3-/-* mutants (Figure 2C,G). *fgf10a* morphants showed a partial loss of Pax2a+ cells, albeit more severe than *fgf3-/-* mutants (Figure 2D,G). In contrast, combined inactivation of Fgf3 and Fgf10a resulted in a complete loss Pax2a expression in the facial placode, and a near complete loss of Pax2a expression in the glossopharyngeal/vagal placode at 24 hpf (Figure 2F,G). In addition to Pax2a, we also observed a loss of *sox3* expression in Fgf3+10a deficient embryos at 24 hpf (Figure S2C,D). Importantly, combined loss of Fgf3+10a did not affect the *foxi1*+ expression domain (marks EB placode precursors; Figure S2A,B), indicating that the requirement for Fgf3 and Fgf10a was distinct from an earlier role of Fgf3+8b during EB placode specification. 

To confirm that this phenotype is not due to developmental delay, we assayed the EB ganglia at 3 days post fertilization (dpf) using expression of TgBAC(*phox2b:EGFP*) [17]. As reported previously, *fgf3-/-* mutants displayed a loss of the glossopharyngeal and three small vagal ganglia, however the facial and large vagal ganglia still formed (Figure 2J and [24]). We observed a complete or almost complete loss (occasionally we observed a few cells in the large vagal ganglion) of the EB ganglia in *fgf3-/-* embryos injected with *fgf10a*-MO (Figure 2M). These data reveal that Fgf3 and Fgf10a cooperate during development of the EB placodes; however their combined activity was not required for the development of EB placode precursors and gross development of the otic vesicle. 

A previous study showed that Fgf signaling was required for anterior identity (marked by expression of *pax5*) of the otic vesicle [45]. Fgf3 was identified as a ligand in part responsible for regionalizing the otic vesicle, however only a partial loss of *pax5* mRNA was observed. Embryos treated with SU5402 showed a complete loss of anterior otic markers, indicating that an additional Fgf also acts with Fgf3 to impart anterior otic identity. We asked whether Fgf10a was another ligand responsible for assigning anterior otic identity. We found that combined inhibition of Fgf3 and Fgf10a resulted in a complete loss of *pax5* expression in the otic vesicle at 25 hpf (Figure S2E-H). While Fgf3 and Fgf10a in combination are not important for early otic placode specification or induction, we conclude that these ligands do play a role later to specify otic axial asymmetry.

### Fgf10a and endodermally derived Fgf3 cooperate during EB placode formation

**Figure 3:**
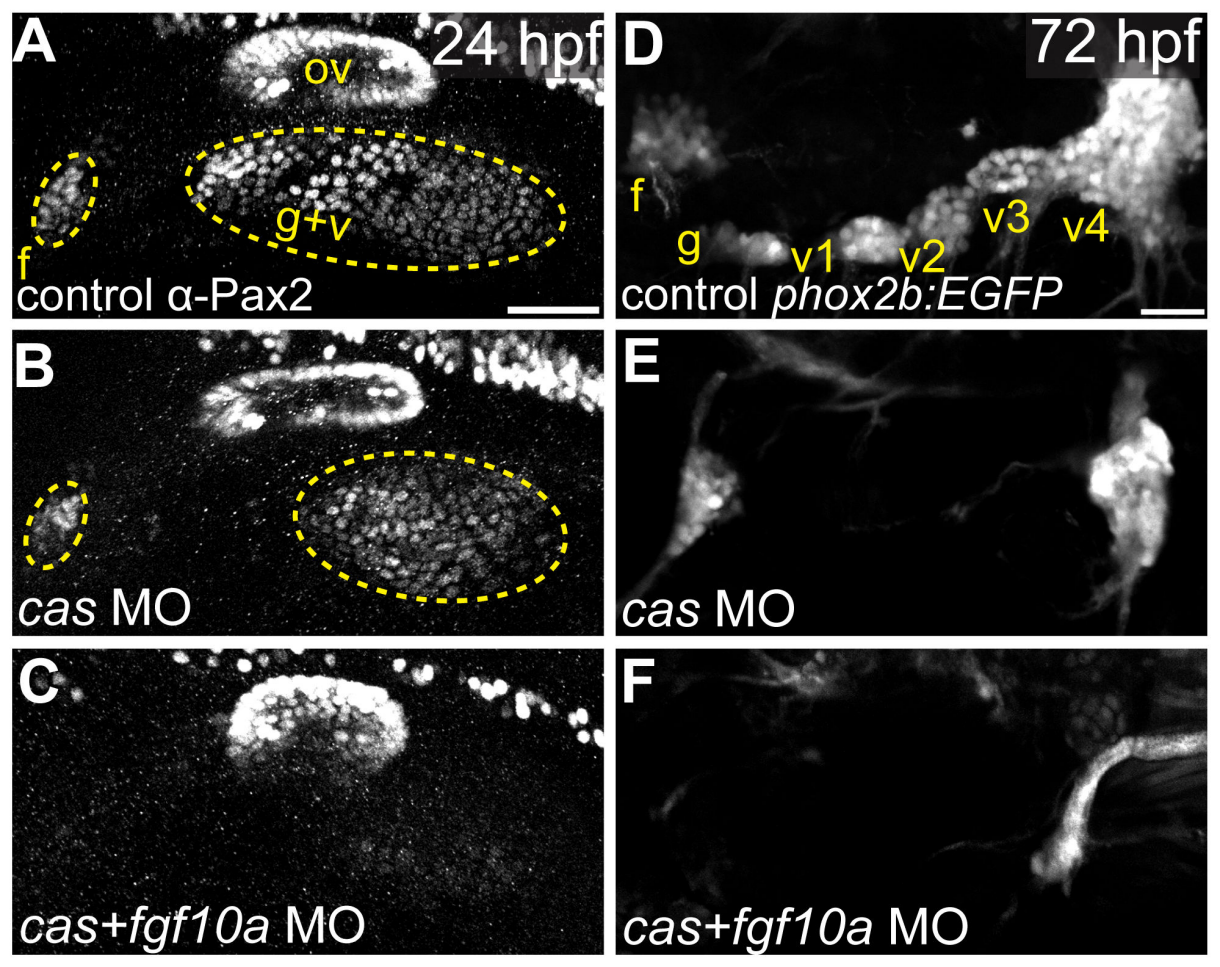
Fgf10a and endodermally derived Fgf3 cooperate during EB placode formation. (**A**-**C**) Confocal projections of Pax2a expression in control (A), *casanova*-MO injected (B), or *casanova/fgf10a*-MO coinjected (C) embryos at 24 hpf. (**D**-**F**) Confocal projections of 72 hpf TgBAC(phox2b:EGFP) in control (D), *casanova*-MO (E), or *casanova/fgf10*-MO (F) embryos. Note a complete loss of Pax2a and EGFP expression in EB placodes and ganglia, respectively, in *casanova*/*fgf10a-MO* embryos (C and F). Abbreviations: f, facial placode (A) or facial ganglia (D); ov, otic vesicle; g+v, glossopharyngeal/vagal placode; *cas*, *casanova*; g, glossopharyngeal ganglia; v1-v3, small vagal ganglia 1-3; v4, large vagal ganglion. Scale bars: 50 µm (A); 25 µm (D).

*fgf3* and *fgf10a* expression analyses revealed that Fgf3 is expressed in the endoderm and Fgf10a is expressed in the anterior portion of the otic vesicle, anterior lateral line, and posterior lateral line (Figure 1). As reported previously [24], genetic ablation of the endoderm using MO against *casanova*, resulted only in a small reduction of the EB placodes as assayed by Pax2a immunostaining at 24hpf (Figure 3A,B). Consistent with these observations, *cas* morphants lacked expression of TgBAC(*phox2b:EGFP*) in the glossopharyngeal and small vagal ganglia, a phenotype very similar to the one observed in the *fgf3-/-* mutant (Figure 3D,E). However, co-injection of MOs against *cas* and *fgf10a* resulted in a near complete loss of the EB placodes (Figure 3C), phenocopying the *fgf3/10a* mutant/morphants. Moreover, we observed a complete loss of the EB ganglia (Figure 3F) in the *cas/fgf10a* double morphants, identical to that observed in the *fgf3/10a* mutant/morphant embryos (Figure 2L). We conclude that endodermally derived Fgf3 in cooperation with Fgf10a is responsible for EB placode maturation.

### The anterior lateral line is the tissue source responsible for facial placode development

**Figure 4:**
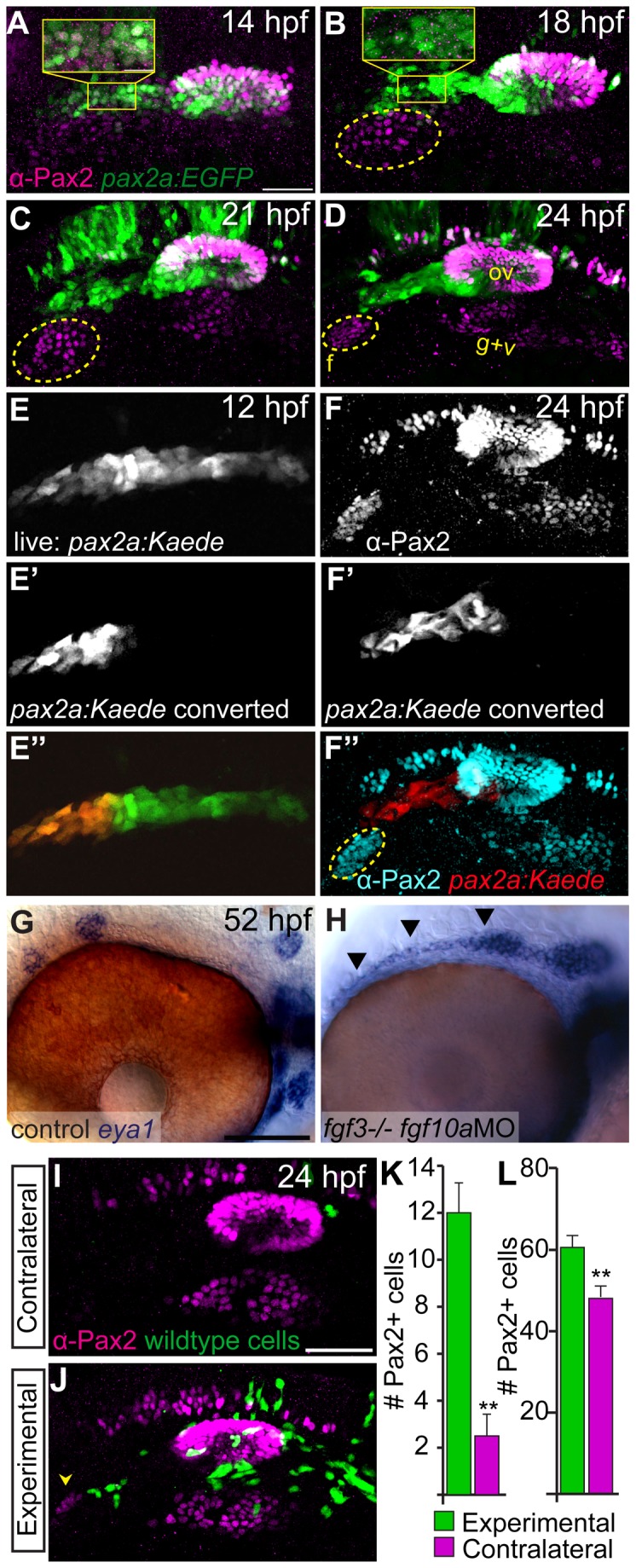
The anterior lateral line is the tissue source of Fgf10a responsible for facial placode development. (**A**-**D**) confocal projections of Tg(pax2a:EGFP) zebrafish embryos (green) analyzed for Pax2a expression (magenta) at 14 (A), 18 (B), 21 (C), and 24 hpf (D). The presumptive facial placode is outlined in yellow as it condenses between 18 and 24 hpf. Insets show co-expression of Pax2a and Tg(pax2a:EGFP) at 14 hpf (A); by 18 hpf, however, Pax2a expression is absent in anterior lateral line precursors, while Tg(pax2a:EGFP) maintains expression in these cells (B). (**E-E**’’) Live confocal projection of a 12 hpf Tg(pax2a:Kaede) zebrafish embryo (E) with the anterior portion of the *pax2a:Kaede*+ domain photoconverted from green to red emission (E’) overlay (E’’). (**F-F**’’) Composite image of the same photoconverted embryo from (E) at 24 hpf analyzed for Pax2a expression (F) and cyan in (F’’) and photoconverted Kaede (F’) and red in (F’’). Note absence of Kaede positive cells in the facial placode. (**G**, **H**) In situ hybridization of *eya1* in 52 hpf zebrafish embryos reveals proper neuromast deposition in control (G) and a failure of deposition and elongation of the anterior lateral line in fgf3-/-;fgf10-MO embryo (H; arrowheads). (**I**, **J**) Lateral views of the 24 hpf fgf3*+10* morphant embryo showing the side that received wild-type donor cells (green) as well as the contralateral control side that did not receive donor cells. Pax2a expression is visualized by immunolabeling (magenta). Note partial rescue of the facial placode when wild-type donor cells were present in the presumptive anterior lateral line (J; arrowhead). (**K**, **L**) Quantification of Pax2a+ cells reveals a significant increase in the number of Pax2a+ cells in the facial (**K**) and glossopharyngeal and vagal placodes (**L**) of the transplanted sides versus contralateral sides (Wilcoxon matched-pairs signed rank test: **P<0.01; error bars: standard error of mean; n=8 embryos). Abbreviations: f, facial placode; g+v glossopharyngeal/vagal placode; ov, otic vesicle. Scale bars: 50 µm (A, I); 25 µm (G).

We next addressed the tissue source of Fgf10a. Our analysis indicated this factor was expressed in the anterior otic vesicle, anterior lateral line and posterior lateral line (Figure 1). For this study we specifically focused on Fgf10a’s expression in the anterior lateral line during EB placode formation, due to the availability of transgenics that label anterior lateral line precursors. The anterior lateral line originates from the anterior portion of the PPA marked by Pax2a expression at 12 hpf [11,12]. The anterior lateral line anlagen can be visualized by Tg(*pax2a:GFP*) which has an expression pattern similar to that of endogenous Pax2a between 12 and 14 hpf (Figure 4A inset and [12]). By 18 hpf, the anterior lateral line down regulates Pax2a, however the Tg(*pax2a:GFP*) maintains EGFP expression in this structure. The anterior lateral line is in close proximity to the condensing facial placode, which begins to express Pax2a at 14 hpf (Figure 4B). Notably, Tg(*pax2a:GFP*) is not expressed in EB placodes likely due to a lack of necessary enhancers (Figure 4D; ref). The anterior lateral line maintains this close association to the facial placode as it matures and condenses between 14 and 24 hpf (Figure 4C,D). 

Given the close association of the facial placode with the anterior PPA, we examined the role of the anterior PPA cells in facial placode development. Fate mapping analysis using a TG(*pax2a:Kaede*), in which the anterior portion of the Pax2a+ domain was photoconverted at 12 hpf (photoconverted, red fluorescent Kaede protein is stable for several days), revealed that this domain did not significantly contribute to the facial placode (Figure 4E, F). However, our previous study clearly demonstrated the anterior PPA was required for proper development of the facial placode, because ablation of these cells greatly reduced the facial placode [12]. Notably, anterior lateral line cells were still present in Fgf3+10a deficient embryos, albeit anterior lateral line neuromasts were not properly patterned in these embryos (Figure 4G,H). These results argue that the anterior lateral line precursors supply a signal necessary for the development of the facial placode.

Our previous fate mapping study showed that the anterior PPA cells contribute to both the anterior lateral line and anterior portion of the otic vesicle [12]. Thus, we asked which of these structures is the source of the signal required for facial placode development. To accomplish this, we transplanted wild-type cells into the presumptive placodal domain of *fgf3+10a* morphants and assayed the resulting mosaic embryos for Pax2a expression at 24 hpf. *fgf3+10a* morphants displayed complete absence of the Pax2a expression in the facial placode, but showed only partial reduction in the glossopharyngeal and vagal placodes when compared to *fgf3* mutants that received *fgf10a* morpholino (compare Figure 2F and Figure 4I), likely due to incomplete knockdown of Fgf3. We observed a partial rescue in embryos that contained wild-type cells medial to the developing facial placode, a presumptive anterior lateral line domain (Figure 4I-L; Wilcoxon matched-pairs signed rank test: **P<0.01; n=8 embryos). The majority of our mosaic experiments contained wild-type cells in both anterior lateral line and the anterior otic vesicle. We did obtain a single embryo that only contained wild-type cells in the anterior portion of the otic vesicle; in that instance, we did not observe rescue of the facial placode. Overall, we did observe a minor increase in Pax2a+ cells in the glossopharyngeal and vagal placodes in our mosaic embryos (Figure 4L). Conversely, a case of wild-type cells in the presumptive anterior lateral line and not the otic vesicle resulted in a partial rescue of the facial placode. Taken together with our lineage, ablation, and Fgf10a expression data, these data argue for a primary role of anterior lateral line derived Fgf10a during facial placode development.

### Fgf3 and Fgf10a are required for placode maturation and neurogenesis

We reasoned that Fgf3+10a activity could be required for one or more of the following steps during EB placode development: 1) placode induction, 2) placode NC interaction required for proper organization of the placodes and subsequent formation of ganglia, and/or 3) placode maturation and neurogenesis. To test the placode induction requirement, we assayed whether Fgf3+10a-deficient embryos displayed thickened ectoderm, a hallmark of placode morphology [46]. TgBAC(*foxi1:d2EGFP*) positive embryos injected with *fgf3+10a*-MOs and uninjected controls were collected at 26 hpf, counterstained with anti-beta-Catenin antibody to visualize cell membranes and then transversely sectioned to reveal epithelial morphology. We observed no difference in the extent of EB *foxi1-EGFP* expression or in the height of ectodermal cells marked by transgene expression between wild-type and *fgf3+10a* deficient embryos (Figure S3A-C). Moreover, neither proliferation nor cell death in EB placode precursors were affected by combined loss of Fgf3 and Fgf10a (Figure S3D,E). From these experiments, we conclude that Fgf3 and Fgf10a do not control changes in placode cell morphology and are not required for the EB placode induction.

Next, we asked if Fgf3 and Fgf10a control development of the chondrogenic neural crest that is required for the proper organization of the EB ganglia [14]. To accomplish this, we visualized placode precursors and NC derived structures using TgBAC(*foxi1:d2EGFP*) and *Tg*(*sox10*(*7.2*)*:mrfp*) in *fgf3+10a* morphants and control embryos at 26 hpf. We observed a disruption of the posterior NC stream that populated branchial arches (arrow heads; Figure S3F,G). This is not surprising, as endoderm secreted *fgf3* is required for pharyngeal arch formation [28]. However, the second NC stream and the posterior most aspect of the third stream were still present in *fgf3+10a*-deficient embryos. Despite their presence, the EB ganglia that develop in close association with these NC streams failed to form in *fgf3+10a*-deficient embryos. This observation indicates that Fgf3 and Fgf10a are unlikely to exert their effect through the disruption of chondrogenic NC. This is consistent with our previous study demonstrating that disruption of chondrogenic NC in zebrafish did not affect formation or neurogenesis of EB placodes, but instead disrupted condensation of the EB ganglia [14].

**Figure 5:**
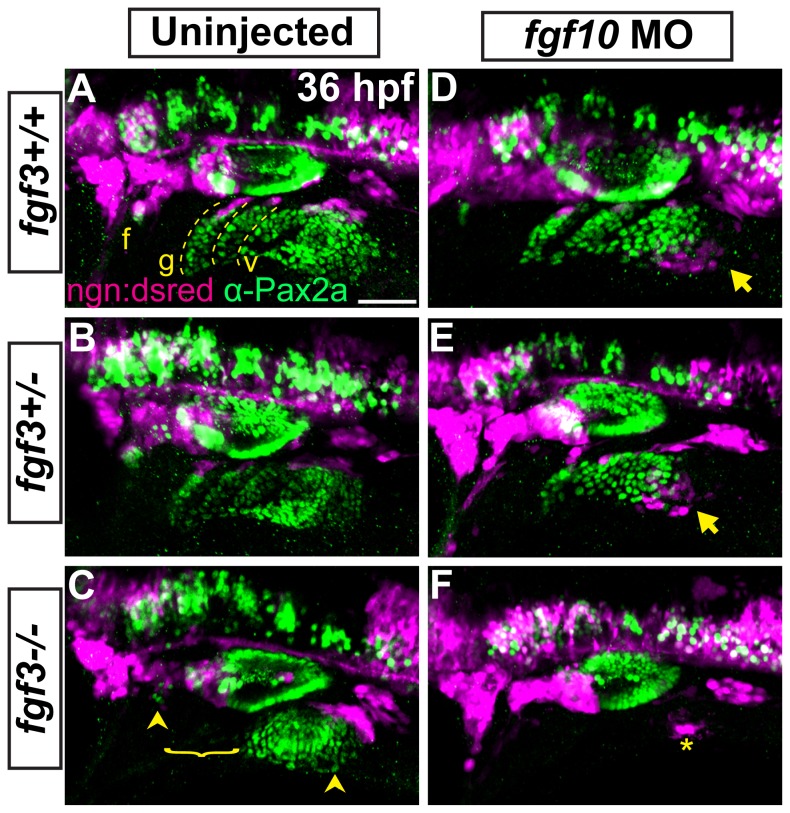
Fgf3 and Fgf10a are required for placode maturation and neurogenesis. (**A**-**F**) Uninjected and *fgf10a*-MO injected progeny from fgf3+/-;TgBAC(neurog1-DSRed) crosses were collected at 36 hpf and immunolabeled for Pax2a and DSRed to visualize EB placodes and migrating neuroblasts, respectively. Wild-type (A) and fgf3+/- (B) panels shows *neurog1:DSRed*+ cells undergoing neurogenesis (magenta) at the dorsal aspect of the mature Pax2a+ EB placodes (green; dotted line). fgf3-/- embryos show a loss of properly formed Pax2a+ EB placodes in the region of the prospective glossopharyngeal and three small vagal ganglia and a concurrent loss of *neurog1:DSRed*+ cells in this region (C; bracket); however, the facial and large vagal placode/ganglia are still present (arrowheads). Analysis of *fgf10a*-MO injected wild-type (D) and fgf3+/- (E) embryos reveal ectopic neurogenesis as marked by *neurog1:DSRed*+ cells ventral to the posterior most aspect of the vagal placode (arrow). *fgf3-/-*;fgf10-MO embryos show a complete loss Pax2a expression in EB placodes and an absence of neurogenesis, except for a few *neurog1:DSRed*+ cells near the region of the large vagal ganglia (F; asterisk). Abbreviation: *ngn:dsred*, TgBAC(neurog1-DSRed); f, facial placode; g, glossopharyngeal placode; v, vagal placodes. Scale bar: 50 µm.

Lastly, we asked if the loss of Fgf3 and Fgf10a inhibits neurogenesis of the EB placodes. To accomplish this we injected *fgf10a*-MO into embryos derived from *fgf3+/-* parents that also carried TgBAC(*neurog1:DSRed*), an early marker of EB placode neurogenesis [17,22]. Embryos were collected at 36 hpf and immunolabled for Pax2a to visualize placodes. In wildtypes, Pax2a+ cells of the EB placodes were arranged in linear fashion (Figure 5A; dashed lines). At the dorsal most aspect of these Pax2a+ arrays, we observed *neurog1:DSRed*+ cells (Figure 5A,B), likely marking delaminating neuroblasts at the medial most aspect of placodes. This is consistent with our previous observations [14] using live analysis of TgBAC(*neuord:EGFP*), demonstrating similar arrangement of neuroblasts in EB placodes. In *fgf3-/-* mutants, we observe a loss of the Pax2a+ structures for the prospective glossopharyngeal and 3 small vagal ganglia concurrent with a loss of neurogenesis (Figure 5C; bracket); however, the facial and large vagal placodes were present and undergoing neurogenesis (Figure 5C; arrowheads). Interestingly in embryos injected with *fgf10a*-MO, while the Pax2a+ placode structures were properly assembled, a subset of EB cells underwent ectopic neurogenesis ventral to the posterior most region of the vagal placode (Figure 5D; arrows). In Fgf3+10a deficient embryos, we observed a complete loss of the Pax2a+ placode structures and a near complete loss of neurogenesis in the region of the nascent EB ganglia, except for a small group of cells postero-ventral to the otic vesicle (Figure 5F; asterisk). These data demonstrate a combined requirement of Fgf3 and Fgf10a during placode maturation and subsequent neurogenesis.

**Figure 6:**
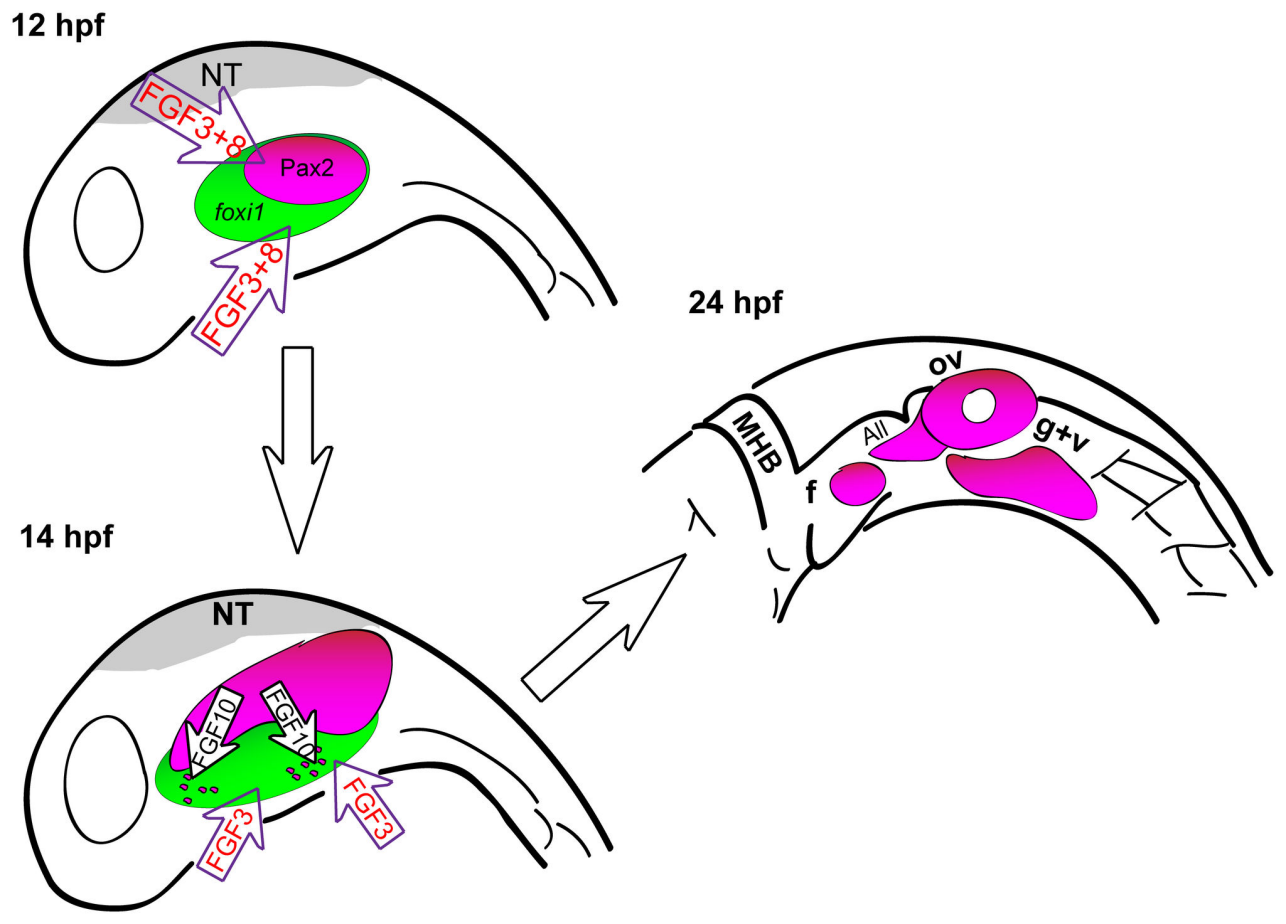
A model for EB placode development in zebrafish. Before 12 hpf, Fgf3 and Fgf8 specify cells of the posterior placodal area that will give rise to the otic and EB placodes. Shortly after, between 14 and 22 hpf, Fgf10a, expressed by the anterior lateral line precursors and the forming otic vesicle, and Fgf3, expressed by the endoderm, are required for EB placode development by promoting the expression of Pax2a and Sox3 in a subset of cells of the Foxi1+ placodal competent ectoderm. By 24 hpf, EB placodes are fully developed. Abbreviations: NT, neural tube; MHB, midbrain hindbrain boundry; f, facial placode; ov, otic vesicle; All, anterior lateral line; g+v, glossopharyngeal/vagal placode.

Our studies highlight the importance of reiterated Fgf signaling to promote specific transcriptional programs necessary during multiple stages of EB placode development. Previous work in zebrafish and other species has shown that Fgf signaling is required to specify the early posterior placodal precursors (Figure 6). After EB placode formation, Fgf3 is required again to initiate neurogenesis of a subset of the EB placodes. However previous studies did not examine other Fgf ligands or a combinatorial requirement of various Fgf ligands during placode maturation and neurogenesis. In this study, we show that Fgf3 and Fgf10a work in concert to promote maturation and subsequent neurogenesis of the EB placodes. Our analyses revealed that Fgf3 and Fgf10a do not control placode cell morphology (Figure S3 A-C). Instead, these factors affect the maturation of the EB placodes by controlling expression of Pax2a and Sox3 required for development and subsequent neurogenesis of the EB placodes.

Whereas Fgf3 signal is endoderm-derived, as previously reported [24], we provide evidence through tissue ablations and transplantation studies, that the developing anterior lateral line is the likely source of Fgf10a responsible for formation of the facial placode (Figure 6). Only a partial rescue of the facial placode was observed in mosaic analysis, a reasonable result due to the additional requirement of Fgf3 from the endoderm. 

We propose that otic and posterior lateral line derived Fgf10a may be similarly required for development of the more posterior, glossopharyngeal and vagal placodes. In support of this role, *fgf10a* transcripts were expressed in the otic placode/vesicle and the posterior lateral line, both of which develop in close proximity to the posterior EB placodes. Our previous study also demonstrated that ablation of the posterior portion of the Pax2a otic domain resulted in a loss of the otic vesicle and a large reduction of the glossopharyngeal/vagal placode [12], supporting the role of the otic placode/vesicle in EB placode development. In addition, our mosaic analysis showed an increase in Pax2a positive cells on the transplanted side relative to the contralateral control side (Figure 4K,L). It may be difficult to completely discriminate whether anterior lateral line expression of Fgf10a is exclusively required for facial placode development, whereas the otic and posterior lateral line foci of Fgf10a are only responsible for the development of glossopharyngeal and vagal placodes. Because both the anterior lateral anlagen and the anterior portion of the otic placode are derived from the same source, rostral part of the PPA [11,12], a vast majority of our mosaic embryos contained cells in these two regions. Nevertheless, together with our ablation data, mosaic analysis argues for a significant contribution of the lateral line derived Fgf10a in the development of the facial placode.

Finally, we cannot exclude the possibility that other otic expressed Fgf factors, in addition to Fgf10a, may play a role in EB placode development. However, our combined knockdown of Fgf3 and Fgf10a resulted in almost complete loss of Pax2a expression and a complete loss of *sox3* in the EB placodes and supports the argument for a major role of Fgf3 and Fgf10a during EB placode development. 

Our finding that the anterior lateral line is the tissue source of Fgf10a required for EB placode development raises an interesting question about the nature of a functionally equivalent signal in higher vertebrates. The anterior and posterior lateral line system is only present in aquatic vertebrates, however a homologous set of EB ganglia is present in terrestrial vertebrates. It is possible that during the course of evolution, different tissues took over as the primary source of necessary signals for EB placode formation. For example, endoderm is thought to be a primary signaling source for EB placode development and subsequent neurogenesis in chick [47]. In mouse, mesoderm derived Fgf3 and Fgf10 are required during early stages of PPA specification [48], and again during otic induction, and subsequent inner ear formation [49]. However, an additional role for these ligands during later stages of EB placode development and subsequent neurogenesis has not been addressed in the mouse. Future studies in other vertebrates will be necessary to determine if a conserved role exists for Fgf3 and Fgf10 during EB placode maturation.

## Supporting Information

Figure S1 **Local Fgf activity is sufficient to expand the facial placode.** (**A**-**E**) Twenty-four hour old zebrafish embryos that received heparin beads soaked in either recombinant Fgf8 (A-C) or BSA (D,E) were immunostained for Pax2a expression and imaged using either transmitted light (shows site of bead implantation in A, D) or confocal microscopy in (B,E; bead is outlined in yellow). Note the expansion of the facial placode (f) near the Fgf8-soaked bead (B) compared to contralateral control of the same embryo (C). (**F**) Quantification of Pax2a+ cells in the facial placode revealed a 2 fold increase in the facial placode in embryos that received an Fgf8 soaked bead compared to the contralateral side (Wilcoxon matched-pairs signed rank test; *P<0.05; error bars: standard error of mean; n=5 embryos/condition).
(TIFF)

Figure S2 **Effects of Fgf3+10a loss on development of EB and otic placodes.** (**A**, **B**) *foxi1* expression detected by in situ hybridization in 16 hpf zebrafish embryos reveals no difference in distribution of EB placode precursors in control (A) and *fgf3-/-*;*fgf10a*-MO (B) conditions. (**C**, **D**) *sox3* expression detected by in situ hybridization in 24 hpf zebrafish embryos. Control shows expression of *sox3* transcripts in the otic vesicle (outlined in yellow), and the EB placodes (C); *sox3* expression is lost in these structures in the *fgf3-/-*;*fgf10a*-MO embryo (D). (**E**-**H**) *pax5* expression detected by in situ hybridization in 25 hpf embryos. Control conditions show expression of *pax5* in the anterior portion of the otic vesicle (E). Whereas only partial loss of *pax5* was observed in *fgf3-/-* (F) or *fgf10a*-MO (G) embryos, complete loss of *pax5* expression was observed in *fgf3-/-*;*fgf10a*-MO embryo (H). Abbreviations: f, facial placode; g+v glossopharyngeal/vagal placode; ov, otic vesicle.
(TIFF)

Figure S3 **Fgf3 and Fgf10a are not required during EB placode induction, proliferation, and survival, but they are required for the EB placode and NC interaction.** (**A**, **B**) Confocal projections of TgBAC(*foxi1:d2EGFP*) (green) 26 hpf zebrafish embryos immunostained for β-Catenin (magenta). Images show unilateral transverse sections at the level of the glossopharyngeal/vagal placode (arrows). Note columnar morphology of the epithelial cells lateral to the otic vesicle in control (A) and *fgf3+10a*-MO embryos (B). (**C**) Average cell height of *foxi1:d2EGFP*+ cells measured in µm was unchanged in fgf3/10a-MO injected embryos compared to controls, measurement non-placodal cells medial to the *foxi1+* cells are significantly shorter (Error bars: standard error of mean. ANOVA multiple comparison with Sidak’s correction; ***P<<0.001; n≥25 cells from 5 individual embryos per condition). (**D**, **E**) Comparison of TUNEL+ cells or PH3+ cells per unit area of the prospective EB placodes between control and *fgf3+10a*-MO injected 18 hpf embryos reveals no change in cell death or proliferation at this stage (n≥8 embryos per condition). (**F**, **G**) Confocal projections of 26hpf embryos derived from crossing *Tg*(*sox10*(*7.2*)*:mrfp*) to TgBAC(*foxi1:d2EGFP*) parents. Control conditions show properly formed branchial arches (F; arrowheads), and mature placodes assembling within corridor like structures (F’, F’’). In *fgf3+10a*-MO embryo, a subset of branchial arches is absent (G; arrowheads); however the anterior and posterior most NC derived structures are still present. Foxi1-positive placodal ectoderm is present, albeit not properly organized at this stage (G’, G’’). Scale bars: 25µm (A, A’); 50µm (F).
(TIF)
