# Supplementary figures and images for: Fgf3 and Fgf10a Work in Concert to Promote Maturation of the Epibranchial Placodes in Zebrafish
Source: PLoS One. 2013 Dec 17;8(12):e85087. doi: 10.1371/journal.pone.0085087 (PMC3866233; doi:10.1371/journal.pone.0085087)

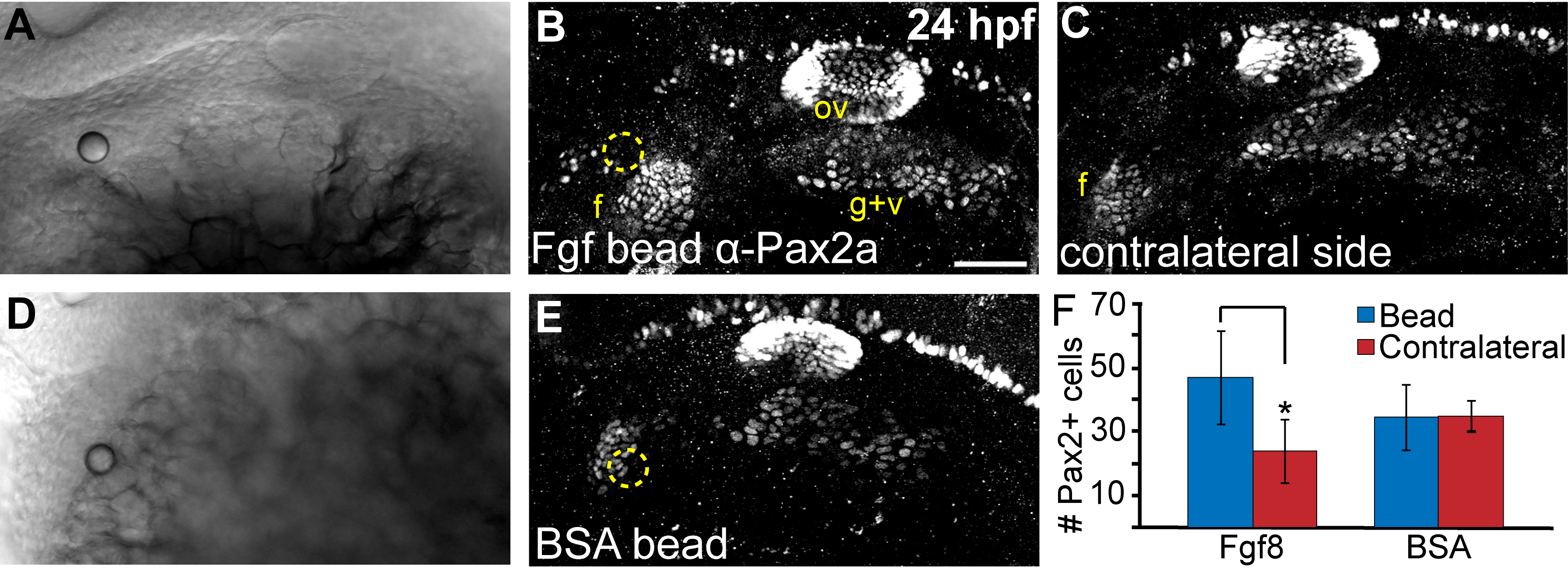

Supplement: Figure S1 — Local Fgf activity is sufficient to expand the facial placode. (A-E) Twenty-four hour old zebrafish embryos that received heparin beads soaked in either recombinant Fgf8 (A-C) or BSA (D,E) were immunostained for Pax2a expression and imaged using either transmitted light (shows site of bead implantation in A, D) or confocal microscopy in (B,E; bead is outlined in yellow). Note the expansion of the facial placode (f) near the Fgf8-soaked bead (B) compared to contralateral control of the same embryo (C). (F) Quantification of Pax2a+ cells in the facial placode revealed a 2 fold increase in the facial placode in embryos that received an Fgf8 soaked bead compared to the contralateral side (Wilcoxon matched-pairs signed rank test; *P<0.05; error bars: standard error of mean; n=5 embryos/condition). (TIFF) [file pone.0085087.s001.tiff]

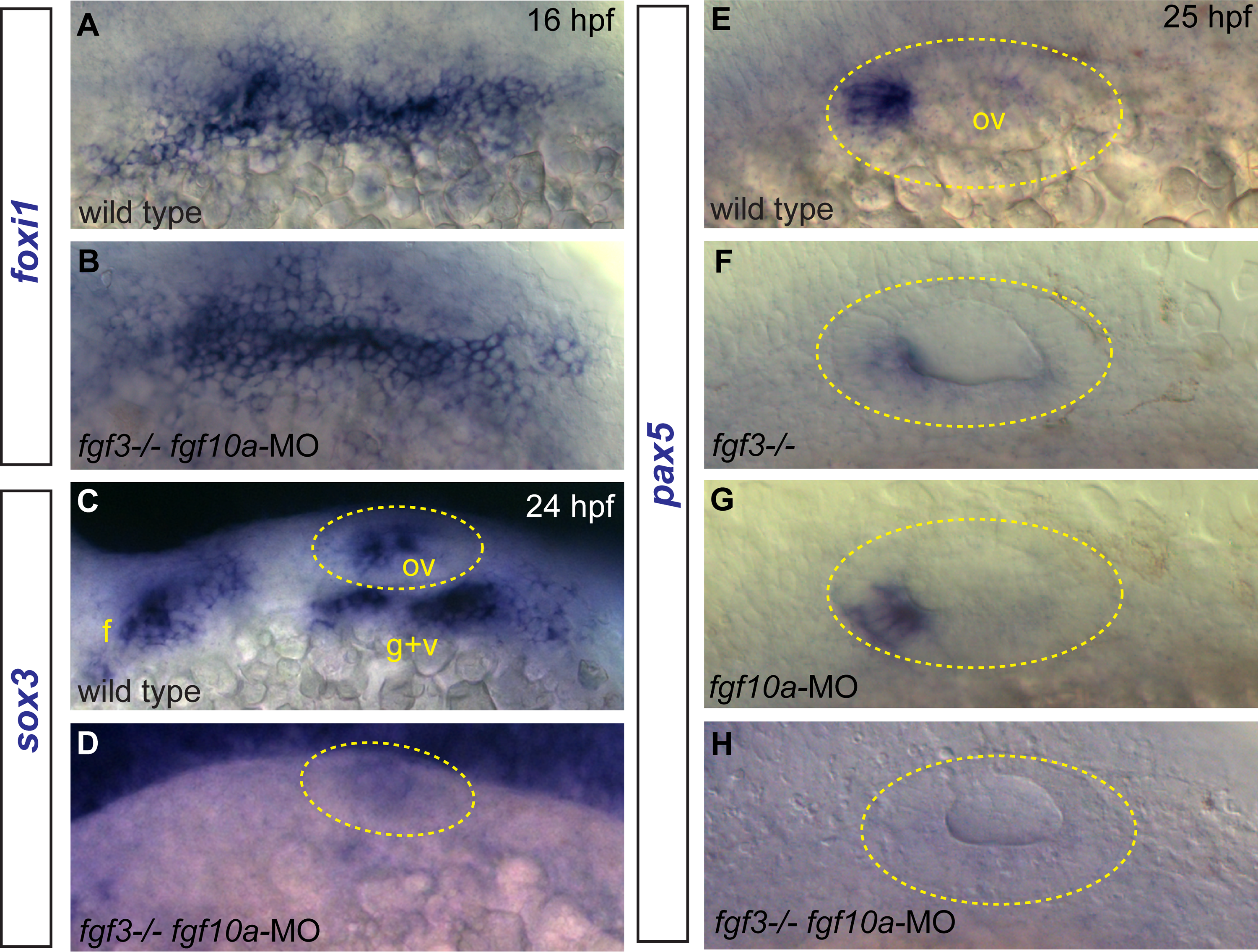

Supplement: Figure S2 — Effects of Fgf3+10a loss on development of EB and otic placodes. (A, B) foxi1 expression detected by in situ hybridization in 16 hpf zebrafish embryos reveals no difference in distribution of EB placode precursors in control (A) and fgf3-/-;fgf10a-MO (B) conditions. (C, D) sox3 expression detected by in situ hybridization in 24 hpf zebrafish embryos. Control shows expression of sox3 transcripts in the otic vesicle (outlined in yellow), and the EB placodes (C); sox3 expression is lost in these structures in the fgf3-/-;fgf10a-MO embryo (D). (E-H) pax5 expression detected by in situ hybridization in 25 hpf embryos. Control conditions show expression of pax5 in the anterior portion of the otic vesicle (E). Whereas only partial loss of pax5 was observed in fgf3-/- (F) or fgf10a-MO (G) embryos, complete loss of pax5 expression was observed in fgf3-/-;fgf10a-MO embryo (H). Abbreviations: f, facial placode; g+v glossopharyngeal/vagal placode; ov, otic vesicle. (TIFF) [file pone.0085087.s002.tiff]

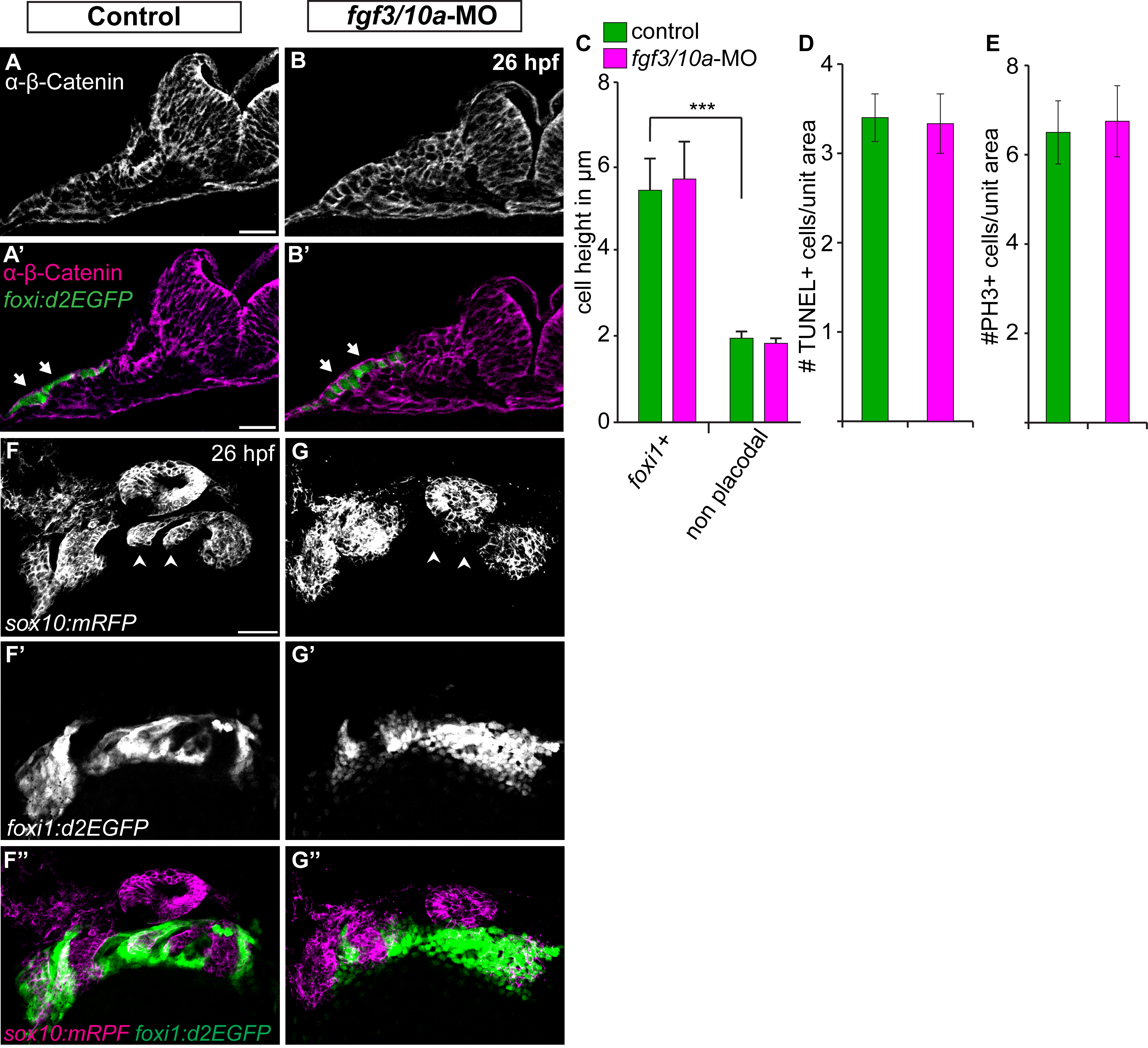

Supplement: Figure S3 — Fgf3 and Fgf10a are not required during EB placode induction, proliferation, and survival, but they are required for the EB placode and NC interaction. (A, B) Confocal projections of TgBAC(foxi1:d2EGFP) (green) 26 hpf zebrafish embryos immunostained for β-Catenin (magenta). Images show unilateral transverse sections at the level of the glossopharyngeal/vagal placode (arrows). Note columnar morphology of the epithelial cells lateral to the otic vesicle in control (A) and fgf3+10a-MO embryos (B). (C) Average cell height of foxi1:d2EGFP+ cells measured in µm was unchanged in fgf3/10a-MO injected embryos compared to controls, measurement non-placodal cells medial to the foxi1+ cells are significantly shorter (Error bars: standard error of mean. ANOVA multiple comparison with Sidak’s correction; ***P<<0.001; n≥25 cells from 5 individual embryos per condition). (D, E) Comparison of TUNEL+ cells or PH3+ cells per unit area of the prospective EB placodes between control and fgf3+10a-MO injected 18 hpf embryos reveals no change in cell death or proliferation at this stage (n≥8 embryos per condition). (F, G) Confocal projections of 26hpf embryos derived from crossing Tg(sox10(7.2):mrfp) to TgBAC(foxi1:d2EGFP) parents. Control conditions show properly formed branchial arches (F; arrowheads), and mature placodes assembling within corridor like structures (F’, F’’). In fgf3+10a-MO embryo, a subset of branchial arches is absent (G; arrowheads); however the anterior and posterior most NC derived structures are still present. Foxi1-positive placodal ectoderm is present, albeit not properly organized at this stage (G’, G’’). Scale bars: 25µm (A, A’); 50µm (F). (TIF) [file pone.0085087.s003.tif]
